# Supplementary material for: An Atypical F-Actin Capping Protein Modulates Cytoskeleton Behaviors Crucial for Trichomonas vaginalis Colonization
Source: Microbiol Spectr. 2023 Jun 13;11(4):e00596-23. doi: 10.1128/spectrum.00596-23 (PMC10434240; doi:10.1128/spectrum.00596-23)
Supplement: Supplemental file 1 — Supplemental material. Download spectrum.00596-23-s0001.docx, DOCX file, 5.0 MB [file spectrum.00596-23-s0001.docx]

**Supplement data and legends**

**Supplementary Figure 1**


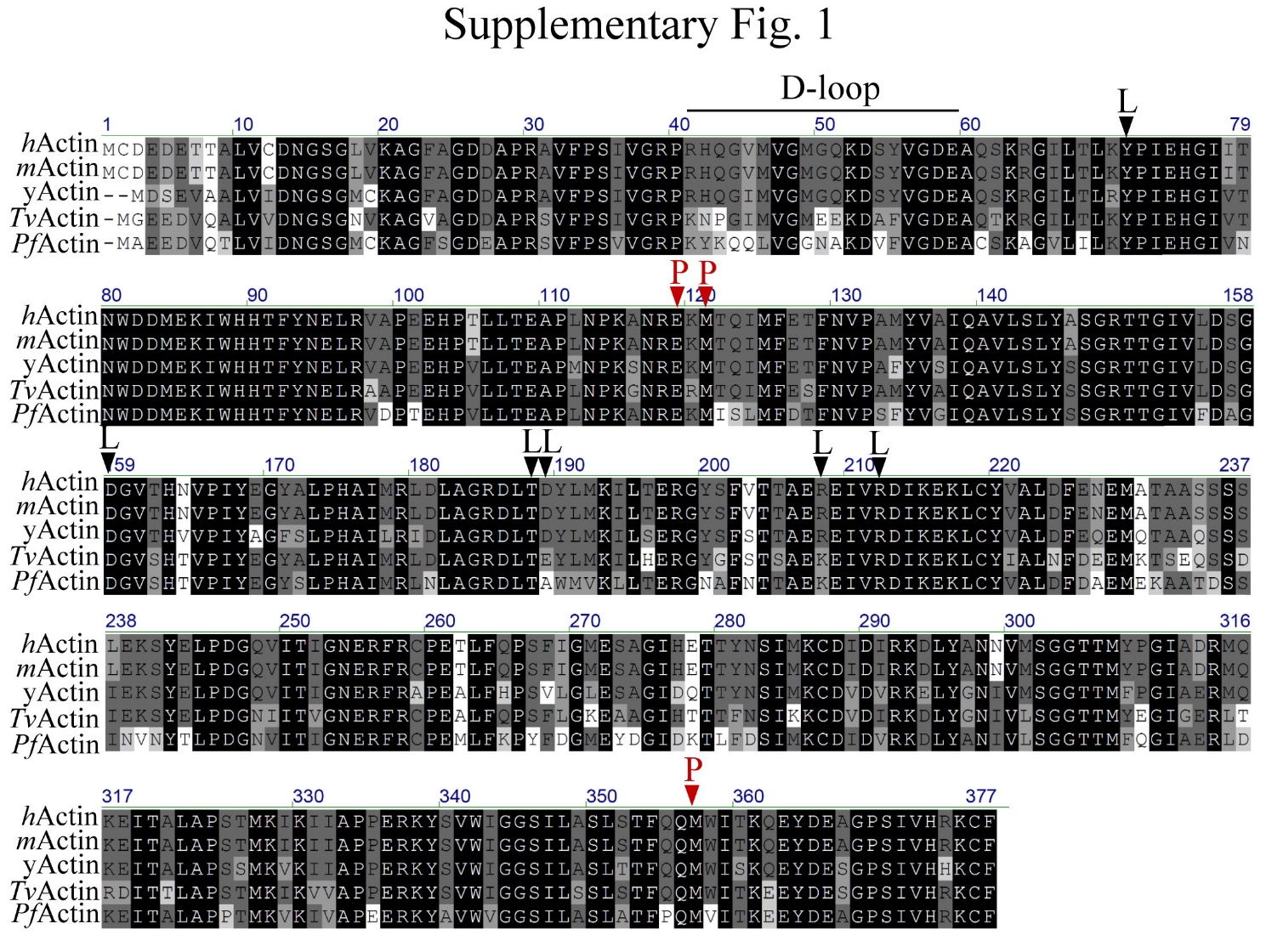


**Supplementary Figure 1. Protein sequence alignment of α-actin.** The full-length protein sequences of α-actin from human (*h*Actin, P68133), mouse (*m*Actin, P68134), yeast (*y*Actin, P60010), *T. vaginalis* (*Tv*Actin, TVAG_337240), and malaria (*Pf*Actin, Q8I4X0) were aligned to show the protein sequence similarity. The conserved amino acid residues are highlighted. The D-loop region and the binding sites of phalloidin or LatB in α-actin are indicated by P or L as shown at the top of sequences, respectively

**Supplementary Figure 2**


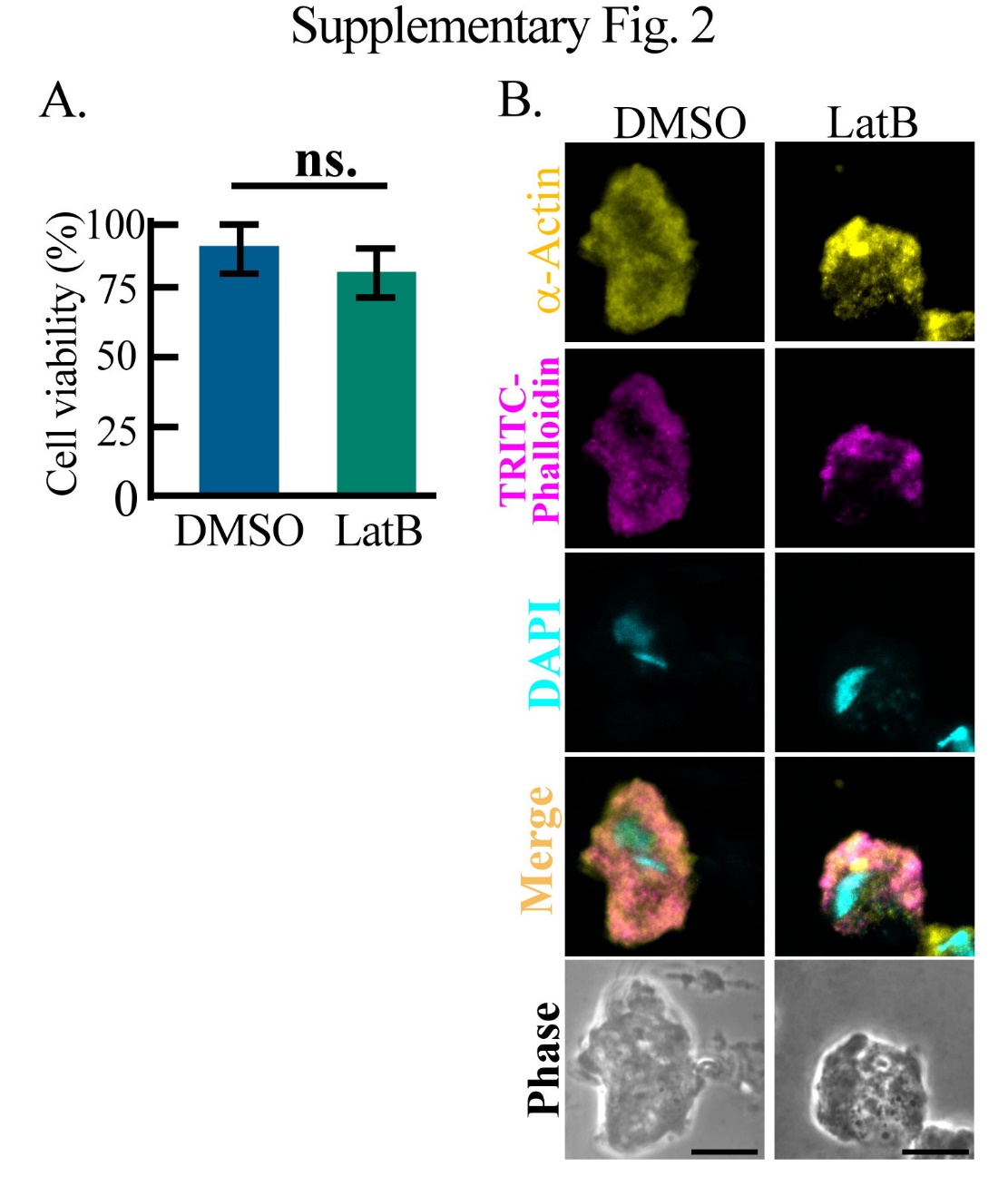


**Supplementary Figure 2. Effects of LatB on *T. vaginalis.*** The TH17 trophozoites were treated with DMSO and LatB for cell viability assay (A.) or co-staining with anti-α-actin and TRITC-Phalloidin to confocal microscopy (B.). For (A.), data in bar graph is presented as mean ± SD. Statistical significance with the p-value for each group of data was analyzed by Student’s t-test as indicated (n=3, *P*< 0.01**, *P*< 0.05*, and ns, no significance). For (B.), the nucleus was stained with DAPI and the cell morphology was recorded with phase-contrast mode. All micrographs were captured in a single Z-slice. Scale bar: 5 μm.

**Supplementary Figure 3**


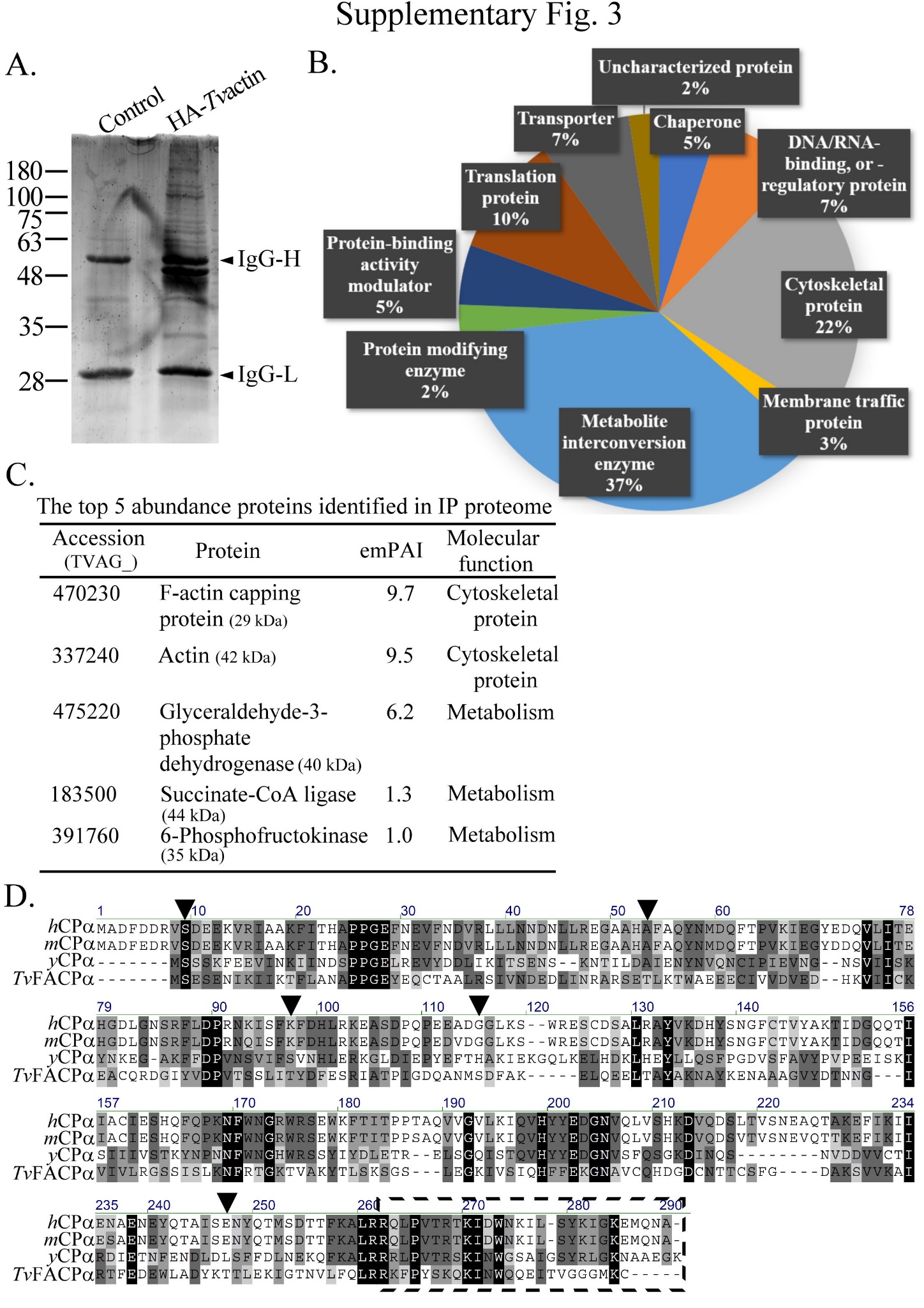


**Supplementary Figure 3. Proteomic identification of actin-binding effectors.** (A.) The immunoprecipitants from non-transgenic control or transgenic TH17 trophozoites overexpressing HA-*Tv*Actin were separated by SDS-PAGE, followed by SYPRO Ruby staining. (B.) In-gel tryptic digests were processed for a label-free quantitative proteomic analysis. The mass identified proteins were classified by function into multiple cellular pathways, including cytoskeleton proteins (22%), chaperones (5%), membrane trafficking (3%), transporter (7%), protein binding (5%), modification (2%), DNA/RNA regulation (7%) and translation (10%), metabolism enzymes (37%), and uncharacterized proteins (2%). (C.) The top five abundant proteins were listed by their emPAI in descending order. All identified proteins were summarized in Table 1. (D.) The full-length protein sequence of *Tv*FACPα was aligned with the CPα from human (*h*CPα, P52907), mouse (*m*CPα, P47753), and yeast (*y*CPα, P28495). The conserved amino acid sequences are highlighted. The predicted Casein kinase II phosphorylation sites are indicated by downward arrowheads, and the actin-binding domain is boxed by a black dashed line.

**Supplementary Figure 4**


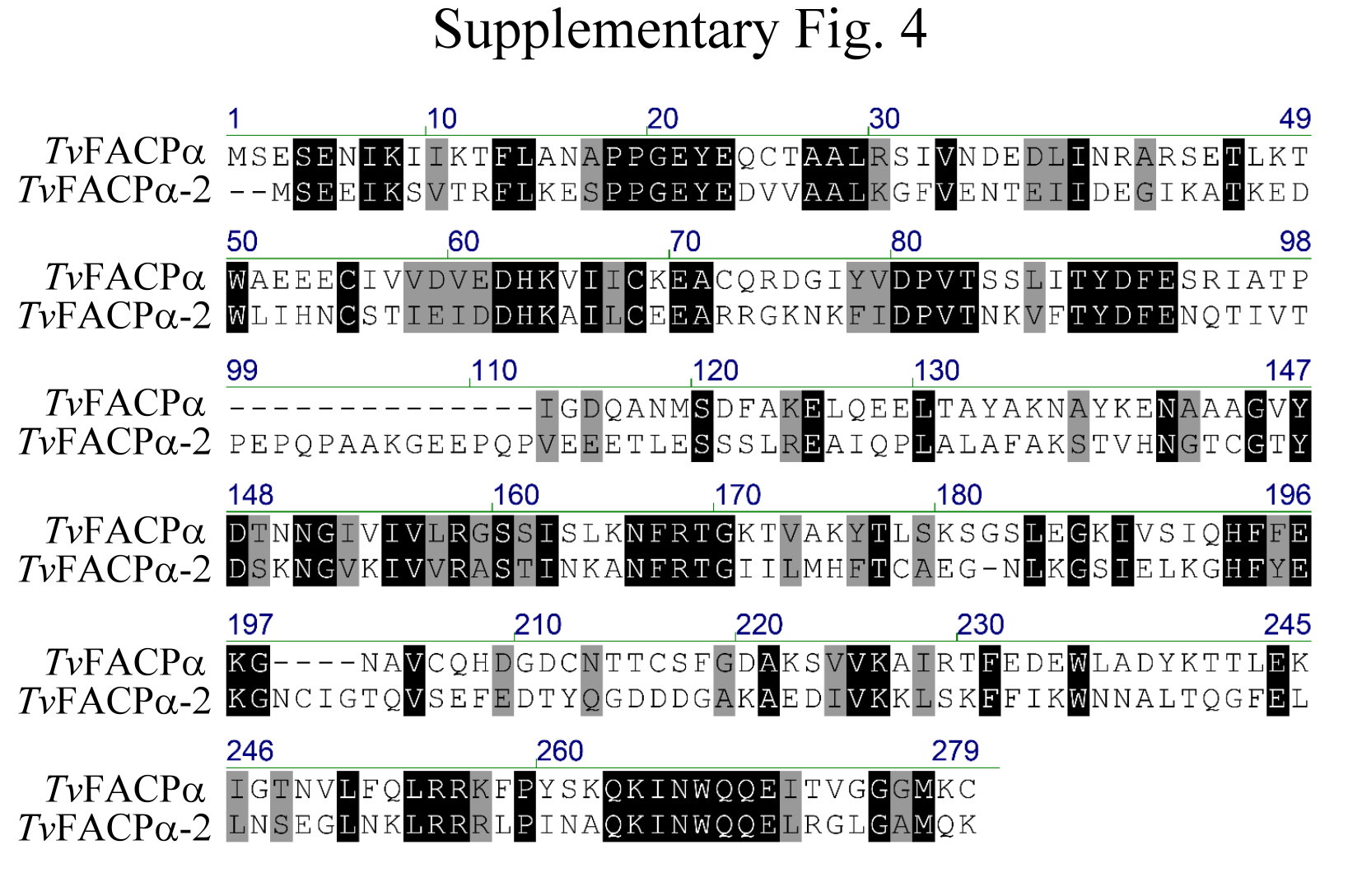


**Supplementary Figure 4. The sequence alignment for *Tv*FACPs in *T. vaginalis.*** The protein sequences of *Tv*FACPα (TVAG_470230) and *Tv*FACPα-2 (TVAG_212270) were aligned. The conserved amino acid residues are highlighted.

**Supplementary Figure 5**


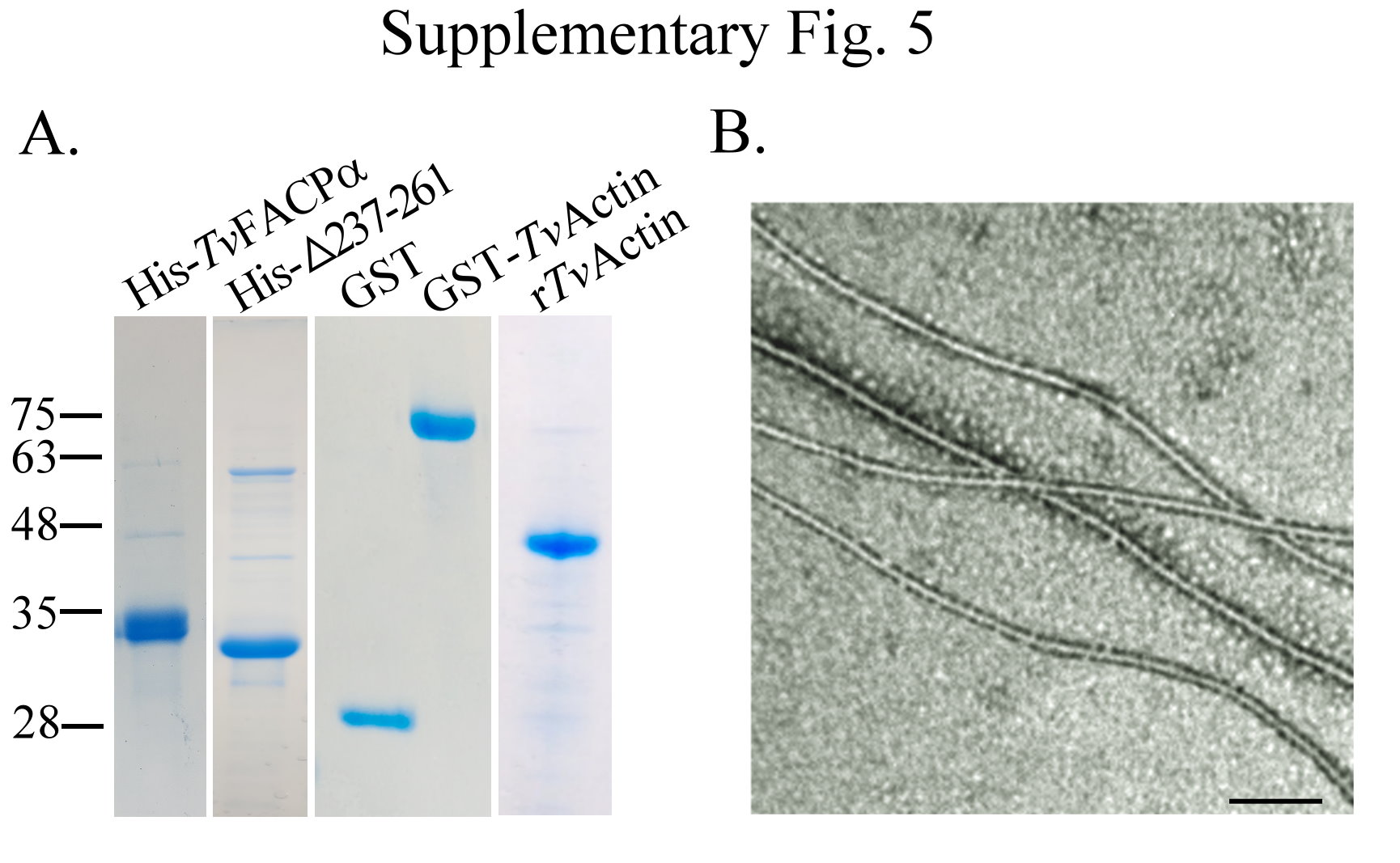


**Supplementary Figure 5. Recombinant protein production for negative-staining TEM.** (A.) The purity of His-*Tv*FACPα, His-△237-261, GST, GST-*Tv*actin, and a tag-less r*Tv*Actin was examined by SDS-PAGE with Coomassie blue staining. (B.) r*Tv*Actin (4 μM) polymerized at room temperature for 1 hr was negatively stained for TEM observation at 40,000× magnification. Scale bar: 0.2 μm.

**Supplementary Figure 6**


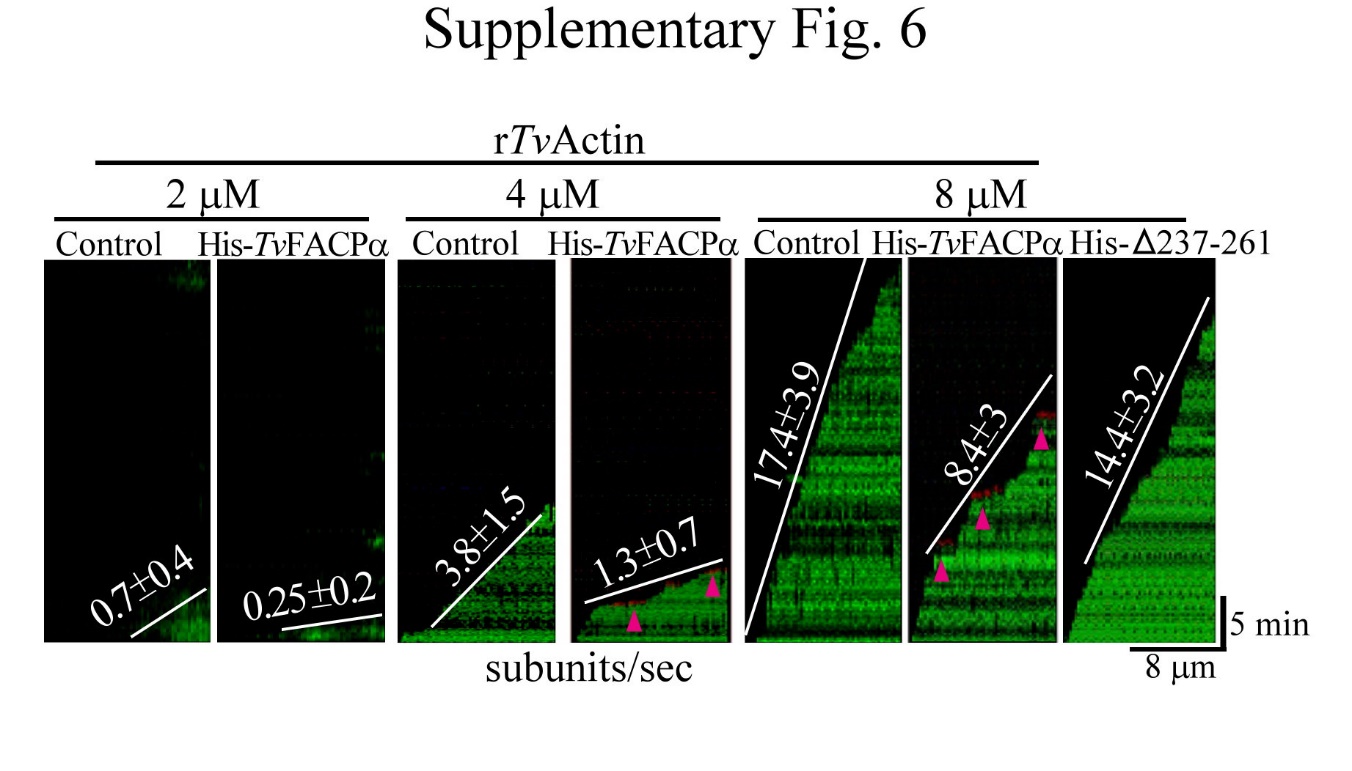


**Supplementary Figure 6. Assembly rates for r*Tv*Actin polymerizations.** The representative raw kymographs showing the assembly rate of r*Tv*Actin at different concentrations were generated by TIRF microscopy (Video 3, 1 μm actin filament= ~370 actin subunits). The magenta arrowheads indicate Alexa Fluor 555-His-*Tv*FACPα detected at the barbed end of a growing actin filament. Assembly rate (submits/sec) is presented as mean ± SD calculated from 25 filaments, from each of two independent protein preparations.

**Supplementary Figure 7**


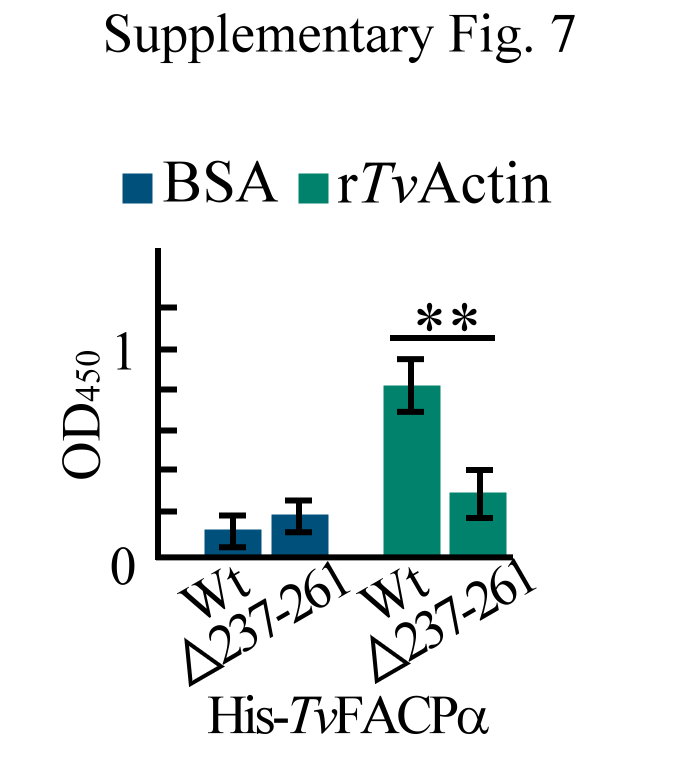


**Supplementary Figure 7. G-actin binding of *Tv*FACPα.** Gel-filtrated monomeric r*Tv*Actin or BSA coated on the 96-well microplate were incubated with equimolar His-*Tv*FACPα or His-△237-261. The sample was reacted with anti-6×His antibody followed by HRP-conjugated secondary antibody. The colorimetric signal was detected spectrophotometrically at OD_450._ The assay was performed in three biological repeats (n=3). Data are presented as mean ± SD. Statistical significance with the p-value was analyzed by Student’s t-test as indicated. (*P<* 0.01**, *P<* 0.05*).

**Supplementary Figure 8**


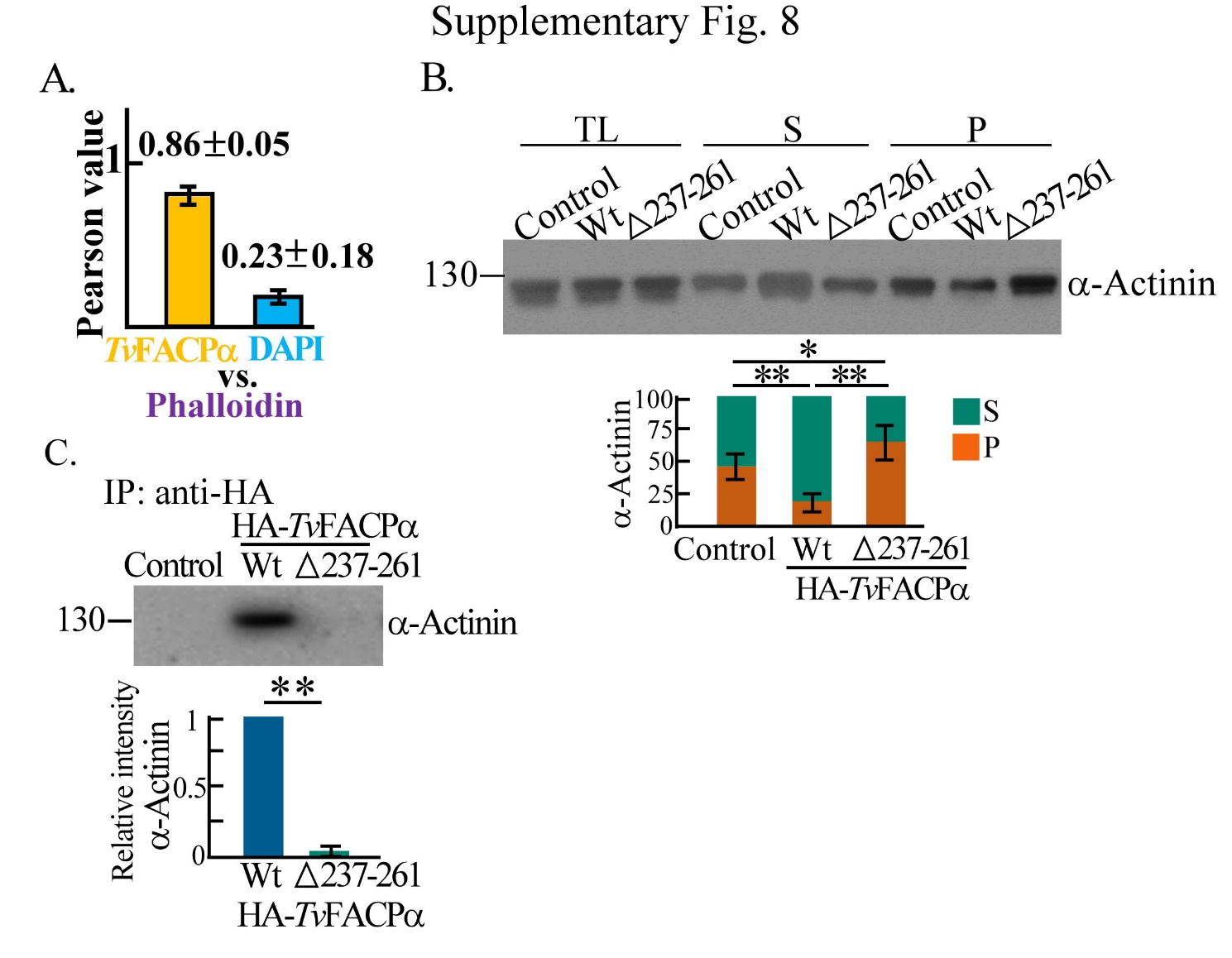


**Supplementary Figure 8. Supplementary data of Figure 5.** (A.) The overall colocalization of phalloidin with *Tv*FACPα or DAPI was evaluated by Pearson correlation coefficient. (B.) and (C.) are the western blotting of α-actinin in Figures 5D and 5E, respectively. Data in the bar graphs are presented as mean ± SD. Statistical significance with the p-value for each group of data was analyzed by Student’s t-test as indicated (n=3, *P*< 0.01**, *P*<0.05*, and ns, no significance).

**Supplementary Figure 9**


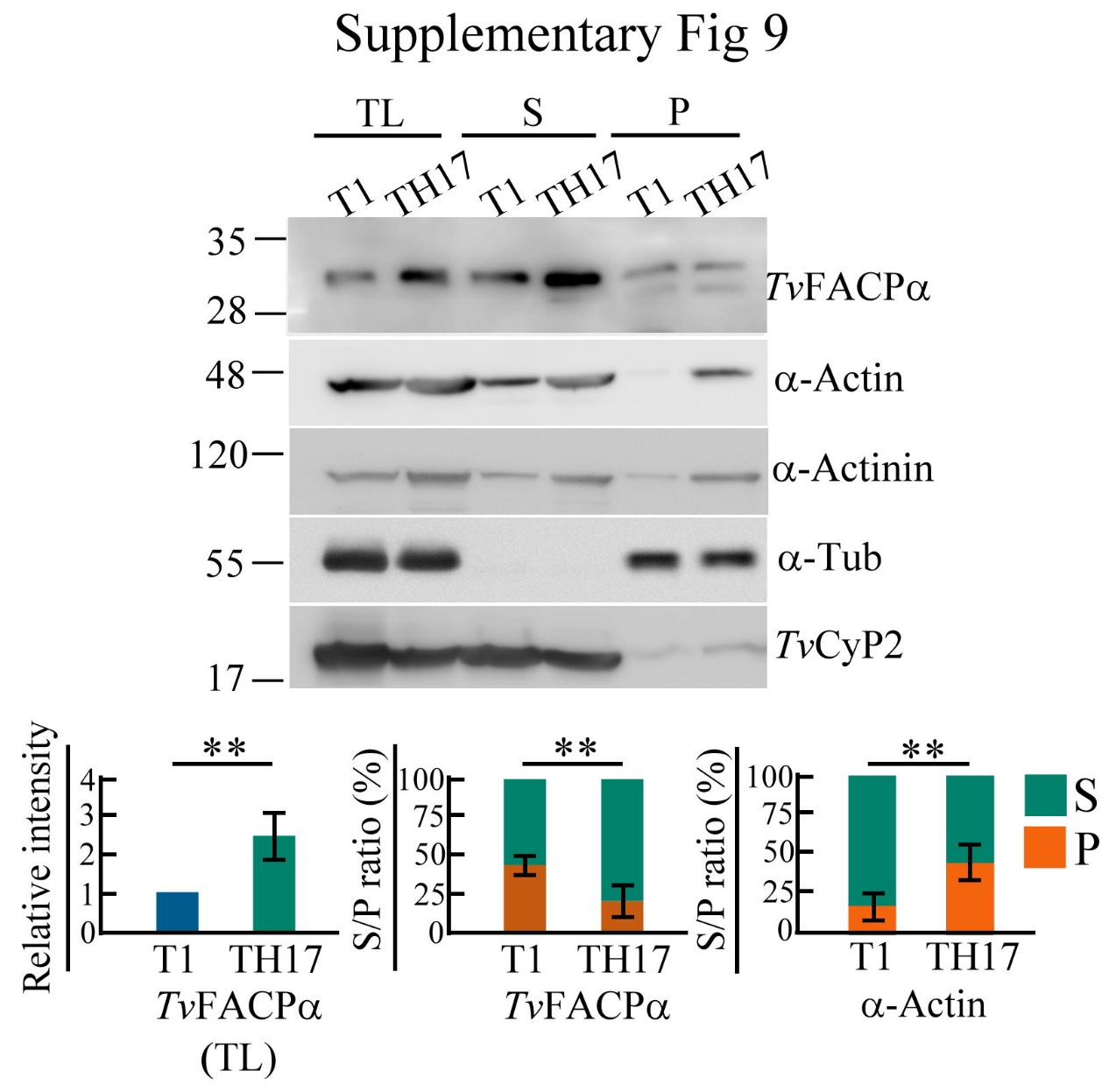


**Supplementary Figure 9. Differential expression of *Tv*FACPα in nonadherent and adherent isolates of *T. vaginalis.*** The protein lysates from Figure 2E were re-examined by western blotting with the anti-*Tv*FACPα antibody. The relative intensity of *Tv*FACPα detected in total lysate, the signal ratios of indicated proteins in the pellet (P) versus supernatant (S) fractions were shown in the bar graphs. The assays were performed in three biological repeats (n=3). Data in bar graphs are presented as mean ± SD. Significance with the p-value is statistically analyzed by Student’s t-test as indicated (n=3, *P<* 0.01**, *P<* 0.05*).

**Supplementary Figure 10**


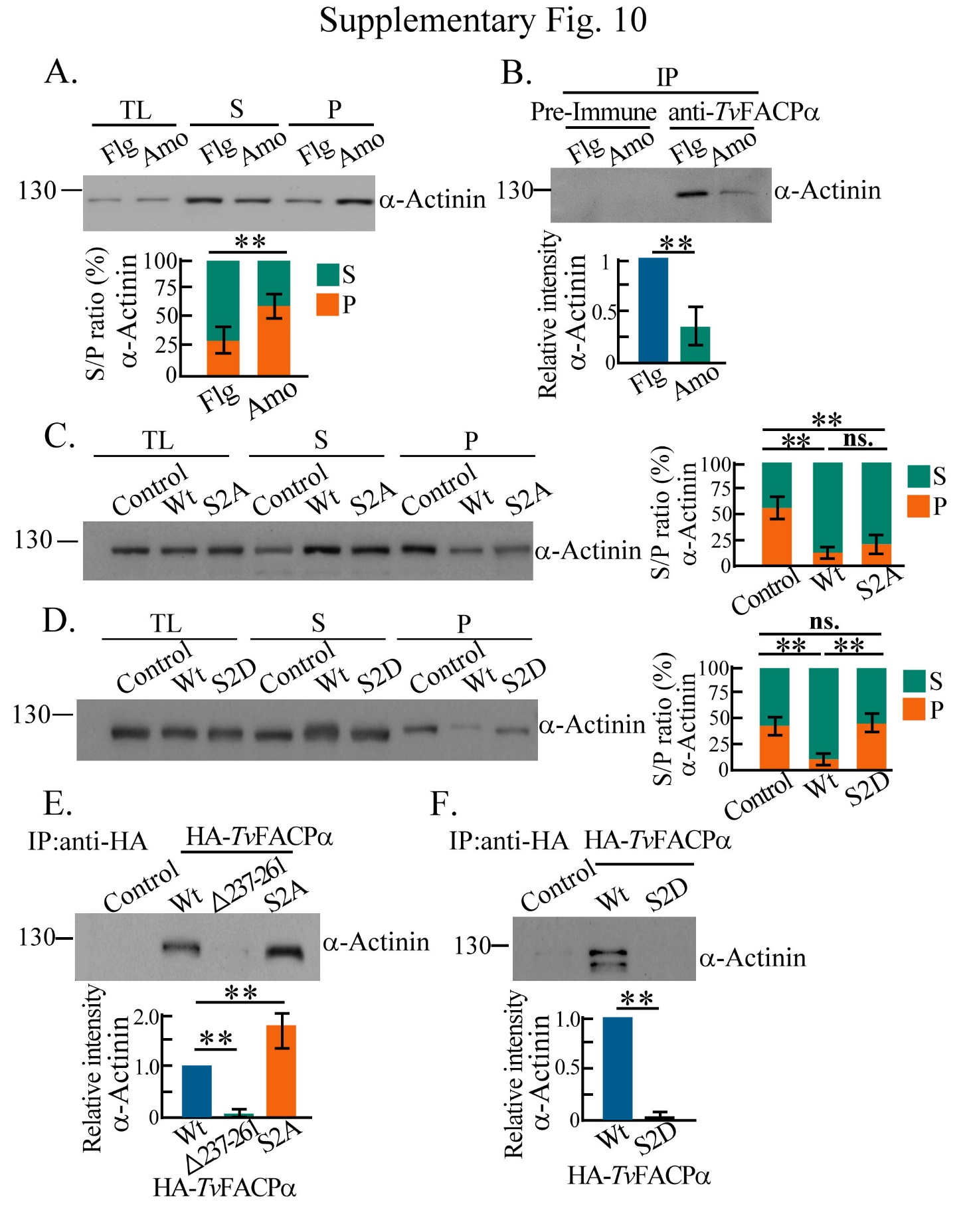


**Supplementary Figure 10. Western blotting detection of α-actinin in Figure 6.** (A) to (F) are the western blotting of α-actinin for Figures 6A to 6F, respectively.

**Supplementary Figure 11**


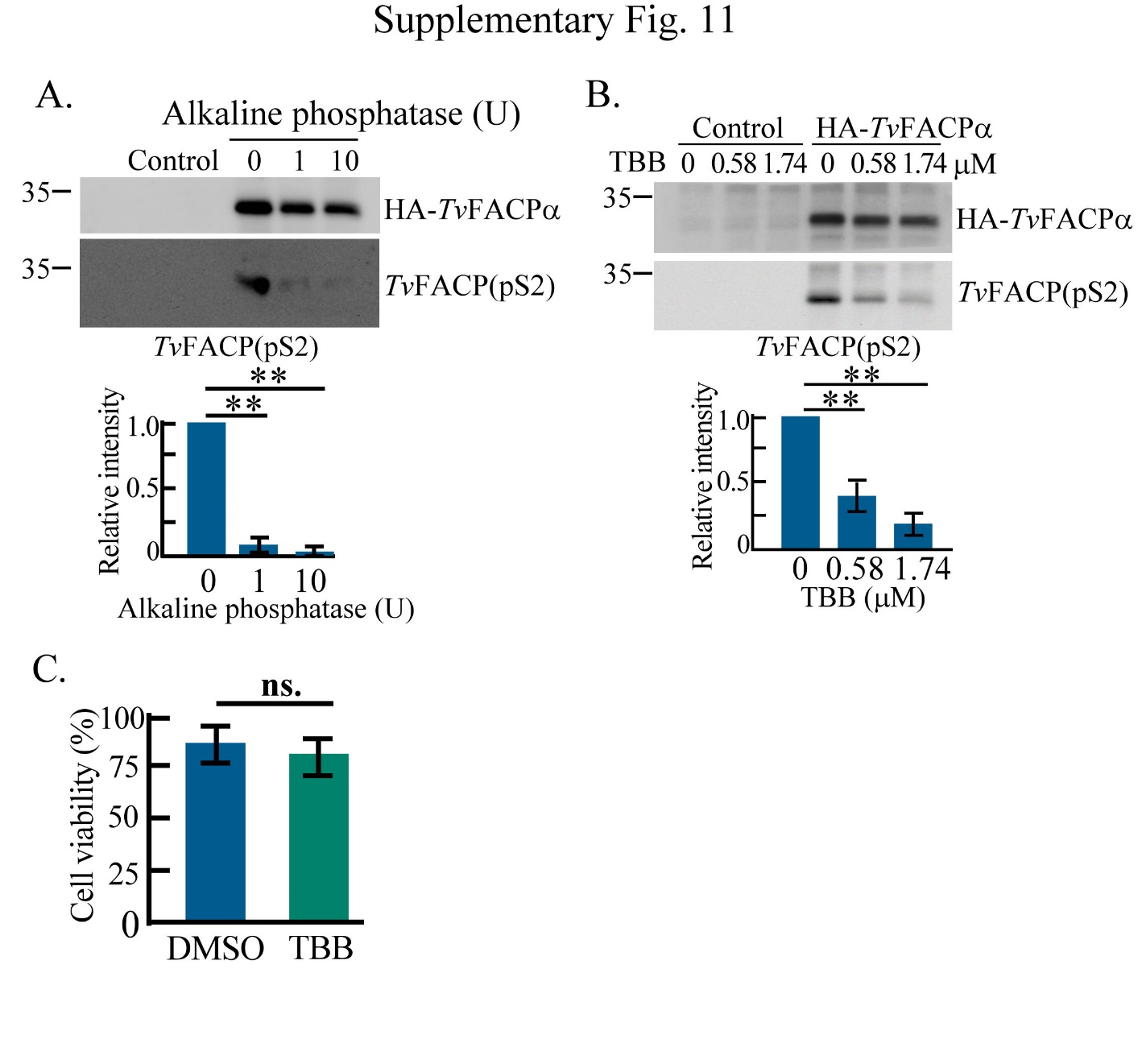


**Supplementary Figure 11*.* *Tv*FACP(pS2) sp** **ecificity test.** (A.) The immunoprecipitated HA-*Tv*FACPα was reacted with different units (U) of alkaline phosphatase at 37℃ for 30 min. (B.) HA-*Tv*FACPα was immunoprecipitated from the HA-*Tv*FACPα transfectant pretreated with different concentrations of TBB. The protein samples were examined by western blotting and the relative intensities were quantified and shown in the bar graphs. (C.) Cell viabilities of trophozoites treated with or without TBB were assessed as shown in the bar graph. The assays were performed in three biological repeats (n=3). Data in bar graphs are presented as mean ± SD. Significant differences with the p-value were statistically analyzed by Student’s t-test as indicated (n=3, *P<* 0.01**, *P<* 0.05*).

**Supplementary Figure 12**


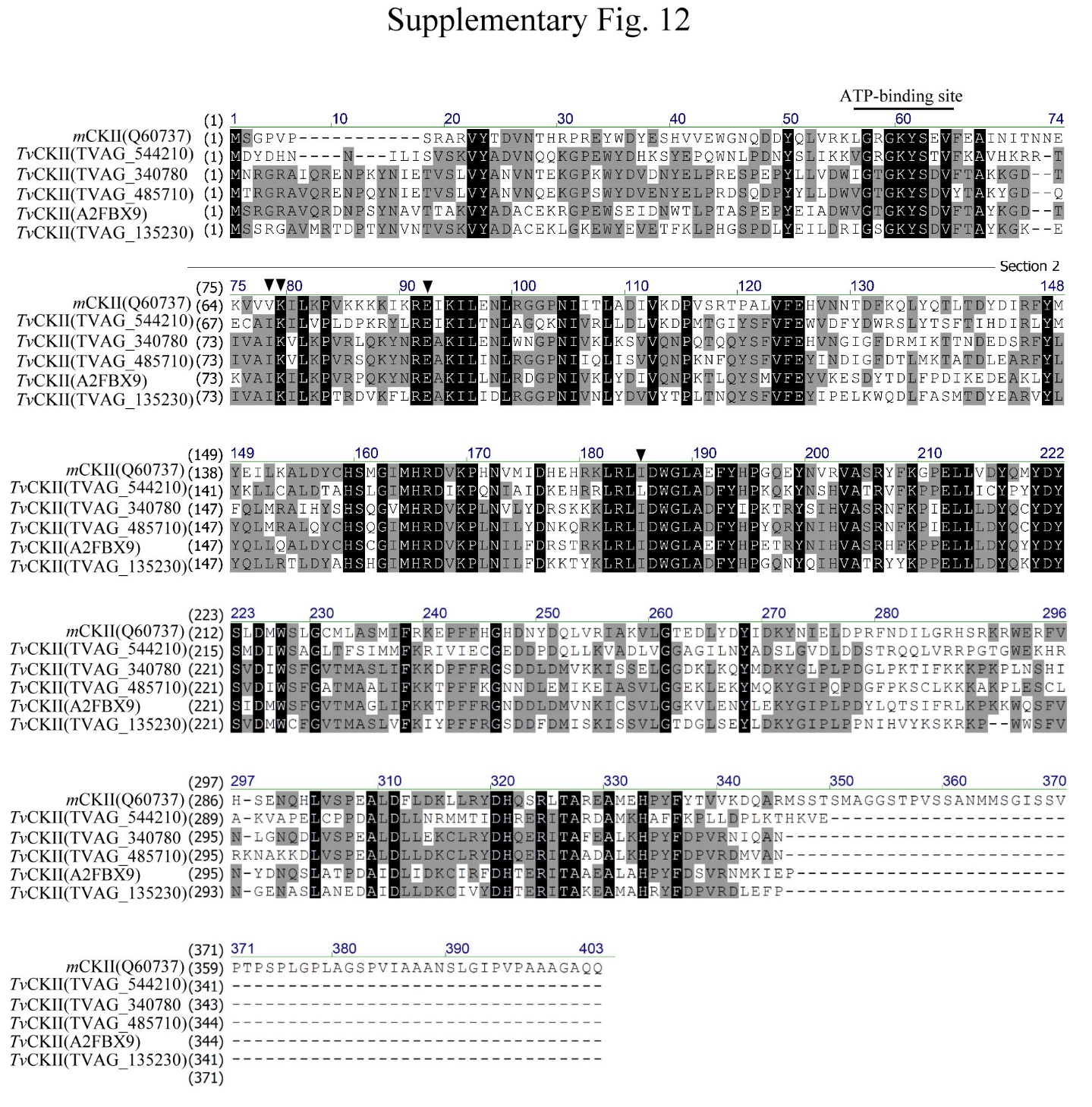


**Supplementary Figure 12. Protein sequence alignment of CKIIα.** The protein sequence of mouse CKIIα (Q60737) was aligned with those of *Tv*CKIIα (TVAG_544210, TVAG_340780, TVAG_485710, A2FBX9, and TVAG_135230). The potential ATP binding site was marked and the amino acid residues involving TBB interaction are indicated by downward arrowheads.

**Supplementary Figure 13**


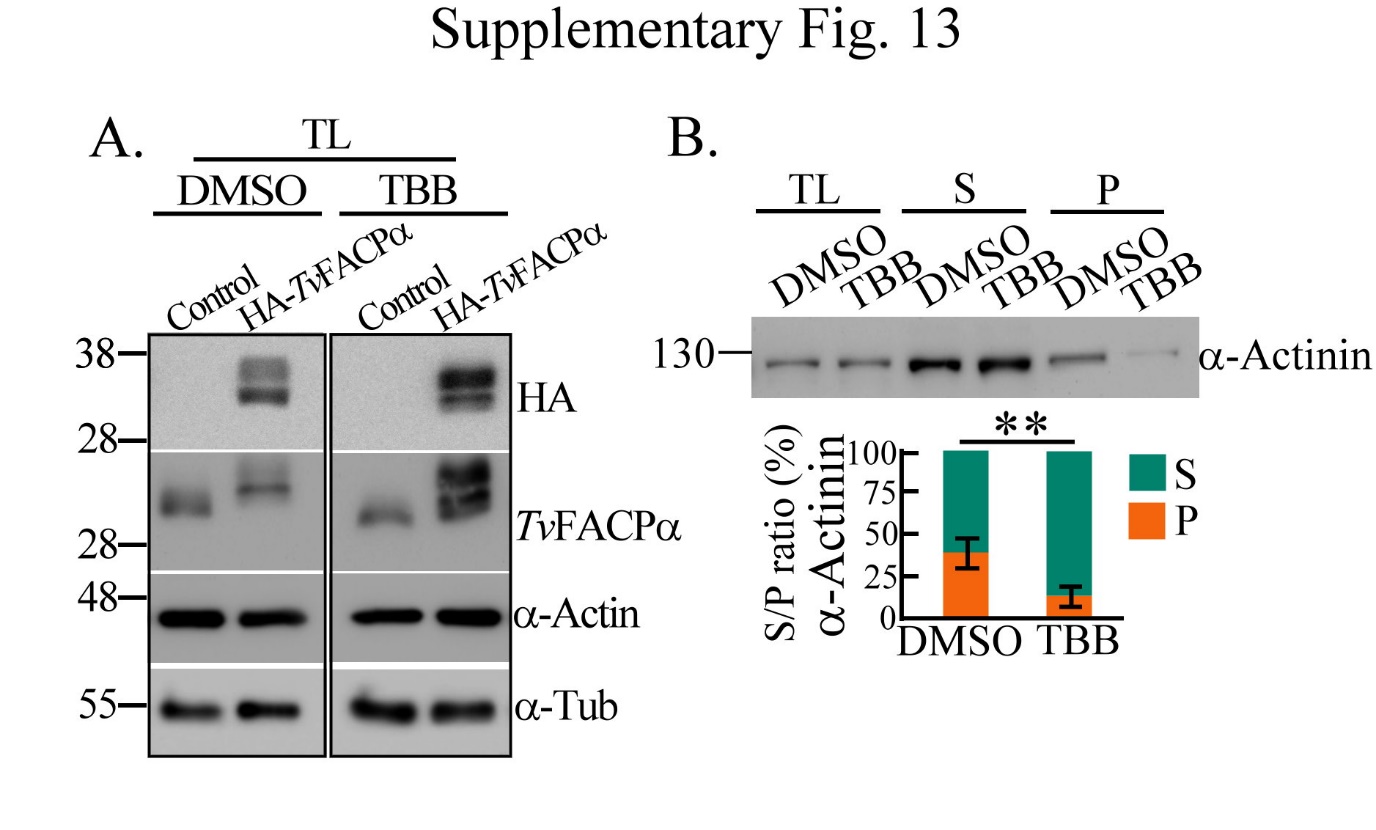


**Supplementary Figure 13. Supplementary western blotting for Figure 7.** (A) Western blots of the total lysates in Figure 7A. (B) Western blotting of α-actinin in Figure 7B.

**Supplementary Figure 14**


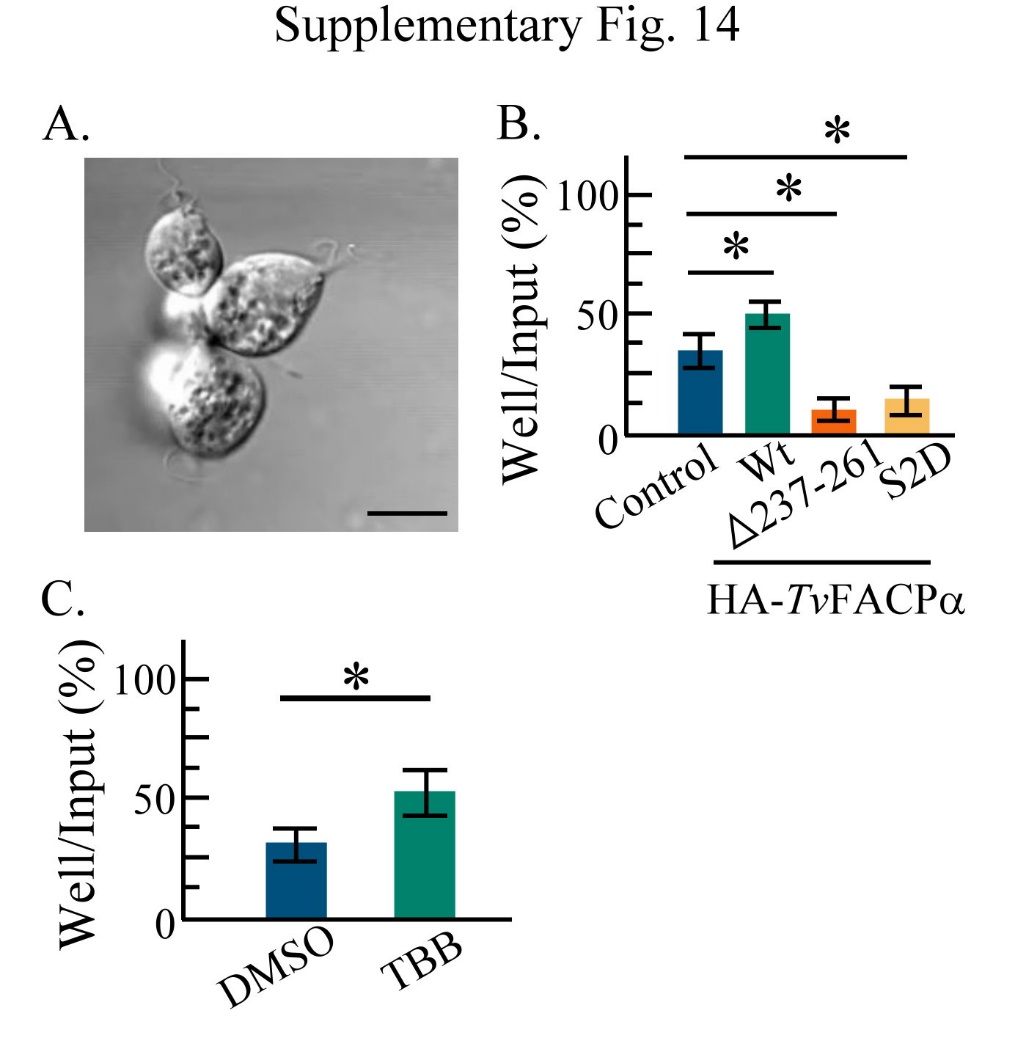


**Supplementary Figure 14. Morphology of tro** **phozoites in the bottom well of the trans-well plate.** (A.) The morphology of trophozoites migrating into the bottom well was recorded by microscopy. The parasites in the bottom well were observed in dominant flagellate trophozoite with clear flagella under our assay conditions. The scale bar represents 5 μm. The data are presented as mean ± SD. Significant differences with the p-value were statistically analyzed by Student’s t-test as indicated (n=3, *P<* 0.01**, *P<* 0.05*).

**Supplementary Figure 15**


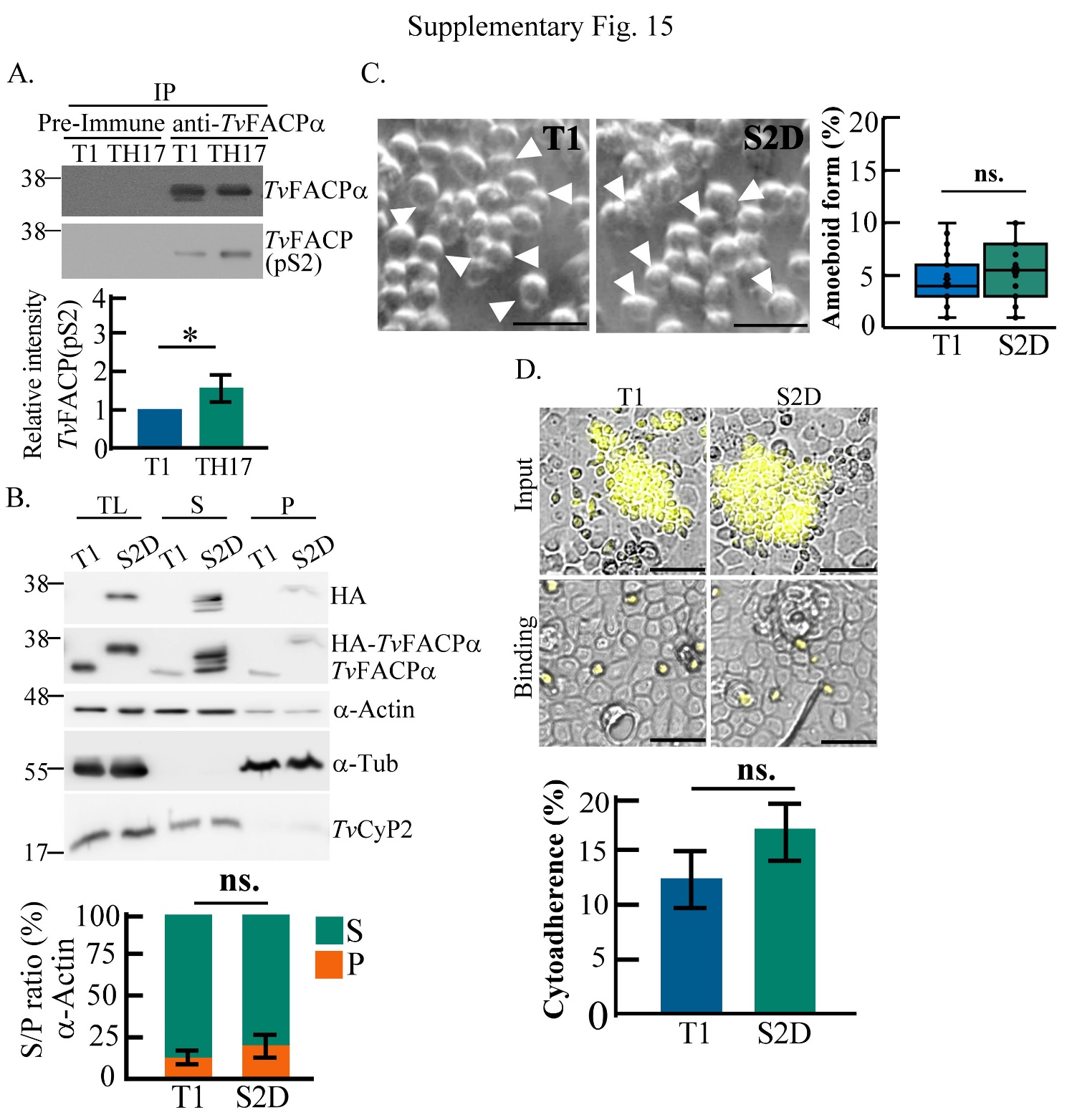


**Supplementary Figure 15. S2D effects in nonadherent T1 isolate.** (A.) The protein lysates from T1 and TH17 trophozoites were immunoprecipitated by anti-*Tv*FACPα antibody for western blotting. (B.) The T1 non-transgenic and transgenic trophozoites overexpressing S2D were fractionated for western blotting. The ratio of α-actin in the pellet (P) and supernatant (S) fractions was quantified as shown in the bar graph. (C.) The morphologies of T1 non-transgenic and transgenic trophozoites overexpressing S2D were observed by phase-contrast microscopy. The proportion of trophozoites in amoeboid form was measured in 600 trophozoites from 12 random microscopic fields as shown in the box and whisker plot. The white arrowheads indicate the flagellate form of trophozoites. Scale bar: 20 μm. (D.) In the binding assay, the conditional trophozoites with CFSE labeling were co-cultured with *h*VECs for 1 hr. The ratio of trophozoites binding versus input was measured as shown in the bar graph. Scale bar: 100 μm.

**Video legends**

**(Please see the attached Video 1)**

**Video 1 Dynamics of amoeboid morphogenesis and migration in the adherent isolate of *T. vaginalis.*** The trophozoites from TH17 adherent isolate were co-cultured with *h*VECs. The dynamics of trophozoite activities were recorded by time-lapse imaging at the capturing rate of one frame per 30 sec over time as defined.

**(Please see the attached Video 2)**

**Video 2 Dynamics of migration in the nonadherent isolate of *T. vaginalis*.** The trophozoites from nonadherent T1 isolate were co-cultured with *h*VECs. The dynamics of trophozoite activities were recorded by time-lapse imaging at the capturing rate of one frame per 30 sec over time as defined.

**(Please see the attached Video 3)**

**Video 3. Observation of r*Tv*Actin polymerization by TIRF microscopy.** 2, 4, 6, and 8 μM of r*Tv*Actin (Alexa Fluor 488 labeled) polymerized with BSA (control), His-*Tv*FACPα, or His-△237-261 (Alexa Fluor 555 labeled) at a molar ratio of 20:1 was recorded by TIRF microscopy. The polymerization images were recorded at a capturing rate of 10 sec per frame over 10 min. Scale bar: 10 μm.

**Table legend**

**(Please see the attached Table 1 file)**

**Table 1. The list of *Tv*Actin-interacted proteins identified by LC-MS/MS.** The proteins identified by mass spectrometry with emPAI value above 0.25 or the peptides specific in the immunoprecipitant of HA-*Tv*Actin were listed.
